# Correction to: Meropenem/Vaborbactam: A Review in Complicated Urinary Tract Infections

**DOI:** 10.1007/s40265-018-0974-7

**Published:** 2018-09-05

**Authors:** Sohita Dhillon

**Affiliations:** Springer, Private Bag 65901, Mairangi Bay, Auckland, 0754 New Zealand

## Correction to: Drugs 2018; 78(12):1259–1270 10.1007/s40265-018-0966-7

The article Meropenem/Vaborbactam: A Review in Complicated Urinary Tract Infections, written by Sohita Dhillon, was originally published Online First without open access. After publication in volume 78, issue 12, pages 1259–1270 Melinta Therapeutics, Inc. requested that the article be Open Choice to make the article an open access publication. Post-publication open access was funded by Melinta Therapeutics, Inc. The article is forthwith distributed under the terms of the Creative Commons Attribution-NonCommercial 4.0 Internationa_l License (http://creativecommons.org/licenses/by-nc/4.0/), which permits any noncommercial use, duplication, adaptation, distribution and reproduction in any medium or format, as long as you give appropriate credit to the original author(s) and the source, provide a link to the Creative Commons license and indicate if changes were made.

The original article was corrected.

